# Comparison of a New Halloysite Based Haemostatic Gauze (SpeedM) With a Kaolin Based Gauze (QuikClot Combat Gauze) in a Standardised Lethal Porcine Model of Uncontrolled Haemorrhage

**DOI:** 10.1016/j.ejvsvf.2026.04.002

**Published:** 2026-04-25

**Authors:** Daniel Hinck, Catharina Gäth, Sabine Wipper, Sebastian Debus

**Affiliations:** a Department for General, Visceral and Vascular Surgery, Klinikum Itzehoe, Itzehoe, Germany; b Department for Trauma and Orthopedics Surgery, Bundeswehrzentralkrankenhaus Koblenz, Koblenz, Germany; c Department for Vascular Surgery, Medical University Hospital Innsbruck, Innsbruck, Austria; d Department for Vascular Medicine, University Heart and Vascular Centre, University Hospital Hamburg-Eppendorf, Hamburg, Germany

**Keywords:** Halloysite, Haemostatic, Haemostatic dressing, Kaolin, Trauma injury, Traumatic bleeding control

## Abstract

**Introduction:**

The study investigates the effectiveness of a newly developed halloysite coated haemostatic gauze (haemostatic gauze (HG), SpeedM) compared with kaolin coated QuikClot Combat Gauze (CG) in a standardised lethal porcine model of arterial haemorrhage. The study aimed to evaluate halloysite's potential as a novel haemostatic agent for traumatic bleeding treatment.

**Method:**

Twenty-one anticoagulated pigs underwent a standardised femoral artery injury under anaesthesia. The animals were randomised to treatment with either HG (*n* = 10) or CG (*n* = 11). After a five minute compression at 200 mmHg, haemostatic performance was assessed based on primary haemostasis, need for secondary application, haemodynamic stability, and survival over a sixty minute observation period after returning to baseline.

**Results:**

Both products achieved primary haemostasis under controlled conditions. Secondary application defined as primary failure was needed in 10% of HG (*n* = 1) and 18% of CG (*n* = 2) but without fatality. The mortality rate owing to unstoppable bleeding defined as final failure was lower in the HG group (10%, *n* = 1) than in the CG group (27%, *n* = 3). One death occurred in each group before a mean arterial pressure ≥60 mmHg was reached. The other two of the three CG fatalities occurred at a mean arterial pressure >60 mmHg.

**Conclusion:**

Although no statistically significant differences were observed between HG and CG regarding mortality (*p* = .59) or re-application rates (*p* = 1.0), the unique physicochemical properties of halloysite may warrant further investigation in future adequately powered studies.

## Highlights

• Exsanguination remains a leading cause of death following trauma.
• Use of haemostatic dressings is recommended as an adjunctive measure for pre-hospital haemorrhage control.
• Halloysite is proven to be biologically safe with high haemostatic activity.
• The nanotubular structure of halloysite may be relevant for haemostatic material development.

## INTRODUCTION

The quality of medical care provided to the polytraumatised patient is reflected in the rate of preventable deaths, defined as trauma fatalities in patients, who would probably have survived if adequate medical care had been provided.^1^, ^2^, ^3^, ^4^, ^5^, ^6^, ^7^ In civilian settings, over 50% of trauma related deaths occur before the patient reaches a medical facility. In military settings, this percentage exceeds 85%.

The leading causes of death remain traumatic brain injury and exsanguination, with mortality rates ranging from 22 – 72% and 13–27%, respectively.^8^, ^9^, ^10^ Death due to haemorrhage is considered significantly more time critical than traumatic brain injury.^9^, ^10^, ^11^

Although the introduction of tourniquets has significantly reduced death from extremity haemorrhage, their effectiveness is limited in cases involving junctional or truncal bleedings.^1^^,^^12^, ^13^, ^14^, ^15^, ^16^ Owing to the limitations of currently available haemostatic products, new formulations have been developed. Second generation products, such as QuikClot Combat Gauze (CG; Teleflex, Morrisville, NC, USA) and Celox (Medtrade, Crewe, UK), have shown superior haemostatic performance compared with first generation agents such as HemCon Chito+ (Tricol Biomedical, Portland, OR, USA) and QuikClot (Z-Medica, Wallingford, CT, USA).^17^ Owing to its structure and properties, the phyllosilicate halloysite has emerged as a promising base material for the development of third generation haemostatic agents. Its naturally occurring nanotubular structure distinguishes it from the plate like kaolin minerals with a larger specific surface area and higher cation exchange capacity. These physicochemical features enhance halloysite's ability to interact with substrates, making it particularly effective for therapeutic applications.

The objective of this proof of concept study was to assess the feasibility of halloysite as a mineral based haemostatic gauze (HG) and to benchmark it against the kaolin based CG.

## MATERIALS AND METHODS

Owing to the similarity of circulatory and organ systems as well as blood volume, pigs are particularly suitable for testing haemostatic agents.^18^ The porcine model used in this study was based on previous investigations by Kheirabadi *et al.*^19^

### Experimental design

Experiments were conducted on an anticoagulated porcine haemorrhage model (*Sus scrofa domesticus*) following approval from the Hamburg Authority for Health and Consumer Protection (Germany, approval number: 112/14). Domestic pigs of both sexes with body weights (BWs) ranging from 60 – 80 kg were used. Animal handling and care adhered to the institutional animal welfare guidelines of the University Medical Centre Hamburg-Eppendorf.

Twenty-one pigs were randomised into two groups after standardised incision of the common femoral artery (CFA): ten animals received treatment with the halloysite based (HG) and eleven animals were treated with the kaolin based gauze (CG).

### Experimental setup

After a twelve hour fasting period, animals were weighed on the day of surgery. Pre-medication was administered *via* intramuscular injection of atropine (0.02 mg/kg BW), ketamine (10 mg/kg BW), and azaperone (8 mg/kg BW). General anaesthesia was induced intravenously with midazolam (0.1 mg/kg BW) and fentanyl (6 μg/kg BW).

Volume controlled mechanical ventilation was performed using a Zeus Infinity Empowered system (Drägerwerk AG & Co. KGaA, Lübeck, Germany). Complete analgesia was achieved *via* continuous intravenous administration of fentanyl at a dose of 6 μg/kg BW per hour. Volatile anaesthesia was maintained using 2% sevoflurane, supplemented with a continuous infusion of midazolam. Haemodynamic stabilisation and fluid resuscitation were achieved through administration of crystalloid solutions (sodium chloride, 5% glucose) and colloid solutions (hydroxyethyl starch [HES]). All animals received a single dose of 10 000 IU of enoxaparin sodium, followed by a continuous infusion of 500 IU/h for the duration of the experiment. At the end of the protocol, animals were humanely euthanised using ebutramide.

A 6 F vascular sheath was placed in the right carotid artery for measuring mean arterial pressure (MAP) and arterial blood gas. Additionally, a Swan-Ganz catheter (Pulsion Medical Systems, Munich, Germany) was inserted *via* the right internal jugular vein to measure central venous pressure, pulmonary artery pressure, and left intraventricular pressure. For additional haemodynamic monitoring, a 4 F PiCCO catheter (Pulsion Medical Systems) was inserted into the right CFA. An 8 F vascular sheath, also placed in the right CFA, allowed the introduction of a 5 F pigtail catheter for blood sampling from the thoracic aorta.

The CFA was dissected *via* a 10 cm skin incision in the left groin region. The exposed artery was then covered with a gauze soaked in 2% lidocaine to prevent vasospasm.

This was followed by a ten minute phase, to stabilise the MAP at ≥ 60 mmHg. Baseline blood samples (full blood count, coagulation profile, and blood gas analysis) were then taken prior to intervention, along with documentation of haemodynamic and respiratory parameters.

**Figure 1:**
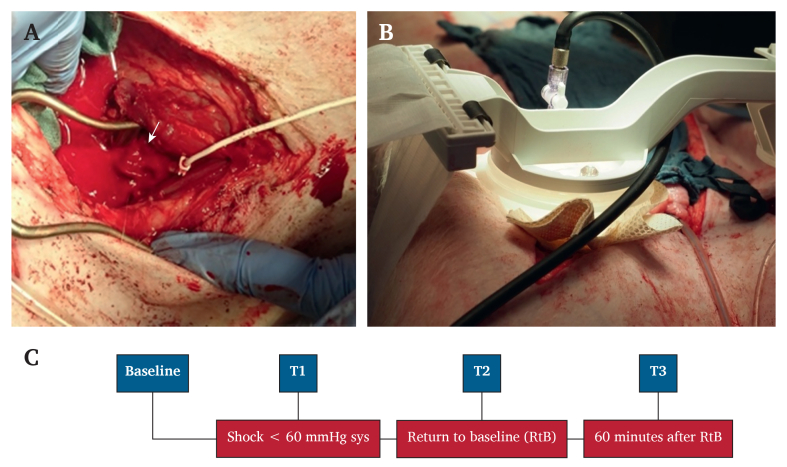
Experimental setup. (A) Exposed common femoral artery with actively bleeding vascular injury (arrow) and Doppler probe placed distal to the arteriotomy for perfusion assessment. (B) Depiction of the test product placed into the groin wound with an inflatable pressure applicator (Femostop). (C) Schematic representation of the measurement protocol showing the time points baseline and T1 – T3. Potential second application of the test product in case of failure (T1b) is not depicted. sys = systolic.

After clamping the exposed CFA, a 4.6 mm arteriotomy was performed using a vascular punch (see Fig. 1A). Following arteriotomy, the proximally and distally placed vascular clamps were removed to allow uncontrolled bleeding until the onset of manifest haemorrhagic shock (systolic blood pressure [SBP] < 60 mmHg).

Additionally, a perfusion Doppler probe (CardioMed Flowmeter, Medi-Stim AS, Oslo, Norway) was positioned distal to the arteriotomy to measure distal perfusion.

Subsequent laboratory testing and documentation of haemodynamic and respiratory parameters were performed (time point T1). Upon confirmation of a SBP <60 mmHg, indicating manifest haemorrhagic shock, the test product (HG or CG) was applied within one minute.

This was followed by a five minute compression at 200 mmHg (cessation of distal perfusion) using an inflatable pressure applicator (Femostop, Abbott, Chicago, IL, USA). No further manipulation was performed.

Fig. 1B illustrates the wound treated with the test product under 200 mmHg compression for five minutes *via* Femostop.

After five minutes, the pressure application was terminated, and haemostasis was assessed (no bleeding from the wound). If bleeding persisted or recurred within fifteen minutes after release of compression (defined as active bleeding from and through the test product constituting primary failure), a second application of the same product was administered, followed by a repeated compression cycle using identical time and pressure parameters. No third application was performed, nor was an application administered fifteen minutes after the first application; ongoing bleeding was then accepted. This was defined as final failure.

In parallel, fluid resuscitation was initiated using 6% HES and an electrolyte solution until baseline values were restored. A MAP >60 mmHg was subsequently maintained by continuous infusion of Ringer's lactate solution.

### Measurement protocol

Haemodynamic and respiratory parameters were recorded at four distinct time points. At each of these time points, haematological samples were also collected.

Initially, following stabilisation of the animal (MAP ≥60 mmHg), the aforementioned parameters were measured and samples collected before femoral incision (baseline).

Time point T1 corresponded to the state of manifest haemorrhagic shock (SBP <60 mmHg), at which the test product was applied, with a potential second application if needed (T1b), followed by time point T2 when baseline MAP was restored. Time point T3 was defined as sixty minutes after MAP had returned to baseline values (see Fig. 1C). At each of these time points, both arterial and venous blood gas analyses were performed. Additionally, blood samples were collected for full blood count and coagulation testing.

### Data analysis

The number of animals was determined based on feasibility considerations and previous experience from comparable studies. Owing to the exploratory proof of concept design, no formal *a priori* power calculation was performed for the final statistical analyses. Primary endpoints were survival time and survival rate.

The Wilcoxon signed rank test was used for statistical analysis of continuously measured parameters, and Fisher's exact test was applied for categorical variables.

All analyses were performed using Microsoft Excel (Redmond, WA, USA). Median values and corresponding interquartile ranges were provided. A *p* value of <.050 was considered statistically significant.

## RESULTS

At baseline, no significant differences were observed in coagulation parameters (platelet count, prothrombin time [Quick], and activated partial thromboplastin time [aPTT]). Haemoglobin (Hb) and haematocrit (Hct) levels were also comparable between groups. A similar pattern was observed for SBP, diastolic blood pressure (DBP), and MAP (Table 1).

**Table 1:** Measured values at baseline and T1 (shock).

Parameter	Baseline			T1 (shock)		
	HG	CG	*p* value	HG	CG	*p* value
Hb (10.8–14.8 g/dL)	8.2 (7.5, 9.1)	7.3 (7.2, 8.7)	>.20	8.3 (8.0, 8.5)	7.9 (7.3, 8.6)	>.20
Hct (33–45%)	26 (23, 28)	21 (22, 27)	>.20	25 (24, 26)	24 (22, 26)	>.20
Platelet count (220–620 × 10^3^/μL)	248 (202, 277)	262 (247, 298)	>.20	254 (234, 299)	255 (233, 312)	>.20
Quick (75–115%)	97 (86, 111)	96 (89, 105)	>.20	77 (68, 81)	89 (82, 99)	.10 < *p* < .20
aPTT (11–15 sec)	99 (88, 122)	101 (85, 114)	>.20	113 (110, 125)	127 (109, 151)	>.20
SBP (100–150 mmHg)	89 (85, 95)	87 (81, 92)	>.20	61 (49, 72)	62 (54, 72)	>.20
MAP (70–90 mmHg)	63 (60, 70)	71 (65, 77)	>.20	35 (34, 38)	41 (30, 53)	>.20
DBP (60–100 mmHg)	51 (46, 54)	53 (49, 61)	>.20	27 (24, 34)	27 (23, 39)	>.20
HR – min^−1^	74 (63, 77)	73 (60, 83)	>.20	95 (71, 123)	82 (76, 114)	>.20
CO (4–8 L/min)	5 (4.5, 5.9)	6.2 (5.1, 7.4)	>.20	3.0 (2.4, 3.9)	4.3 (3.7, 5.5)	.005 < *p* < .010

Depiction at the time of baseline and shock (T1) for Hb and Hct, as well as coagulation parameters including platelet count, Quick, and aPTT. Haemodynamic parameters assessed included SBP, MAP, DBP, and CO). At baseline, values were measured before the induction of hypovolaemic shock (shock). At shock, values were measured before application of the test product and following induction of hypovolaemic shock. Note that a decrease in the Quick value and an increase in aPTT indicated the presence of trauma induced coagulopathy in both groups: HG (SpeedM) and CG (QuikClot Combat Gauze). Data are presented as reference range, median, and interquartile range. aPTT = activated partial thromboplastin time; CG = Combat Gauze; CO = cardiac output; DBP = diastolic blood pressure; Hb = haemoglobin; Hct = haematocrit; HG = haemostatic gauze; HR = heart rate; MAP = mean arterial pressure; SBP = systolic blood pressure.

Table 1 also presents the values at shock induction (T1). Haemodynamic parameters, SBP, MAP, DBP, and heart rate, reflect a state of manifest hypovolaemic shock at time point T1. The shock index (heart rate/SBP) exceeded 1.0 in both groups, which per definition corresponds to severe shock.

No statistically significant differences between the groups were observed for the aforementioned parameters. Similarly, there were no significant differences in Hb, Hct, or coagulation parameters including platelet count, Quick, and aPTT.

However, compared with baseline, the Quick (percentage) value decreased (HG: 97 [86, 111] to 77 [68, 81]; CG: 96 [89, 105] to 89 [82, 99]) and the aPTT(s) was prolonged (HG: 99 [88, 122] to 113 [110, 125]; CG: 101 [85, 114] to 127 [109, 151]), indicating the development of trauma induced coagulopathy.

Following the T1 measurement and application of the test product, substitution with 6% HES was initiated to increase blood pressure. The target was to achieve a MAP ≥60 mmHg or a return to baseline levels.

The obtained haemodynamic and haematological values at the point of return to baseline (T2) and again after sixty minutes (T3) are shown in Table 2.

**Table 2:** Measured values at the time of return to baseline (T2) and sixty minutes (T3) after return to baseline.

Parameter	T2			T3		
	HG	CG	*p* value	HG	CG	*p* value
Hb – g/dL	5.4 (5.3, 5.7)	8.2 (7.4, 9.7)	.010 < *p* < .020	5.6 (5.3, 5.9)	7.6 (7.0, 8.2)	.010 < *p* < .020
Hct – %	17 (16, 18)	25 (23, 30)	.020 < *p* < .050	17 (16, 18)	23 (21, 25)	.010 < *p* < .020
Platelet count – ×10^3^/μL	230 (199, 236)	235 (211, 269)	>.200	214 (190, 241)	228 (206, 266)	>.20
Quick – %	78 (71, 84)	87 (75, 95)	.10 < *p* < .20	76 (69, 91)	74 (62, 89)	>0.20
aPTT – sec	84 (74, 109)	73 (58, 92)	>.20	86 (71, 111)	60 (52, 79)	>0.20
SBP – mmHg	94 (81, 96)	94 (85, 106)	>.20	93 (77, 94)	89 (83, 111)	>0.20
MAP – mmHg	61 (55, 66)	83 (68, 85)	<.001	60 (52, 64)	77 (73, 80)	.005 < *p* < .010
DBP – mmHg	42 (40, 43)	62 (55, 68)	.020 < *p* < .050	39 (33, 47)	64 (56, 67)	.005 < *p* < .010
HR – min^−1^	79 (72,97)	88 (85, 119)	.10 < *p* < .20	79 (71, 102)	85 (79, 107)	>0.20
CO – L/min	6.3 (5.8, 7.4)	7.6 (6.6, 8.6)	>.20	5.6 (5.2, 6.0)	7.4 (6.7, 8.3)	>0.20

Depiction of values at the time point of return to baseline (T2) and sixty minutes after return to baseline (T3) for Hb and Hct, coagulation parameters including platelet count, Quick, and aPTT, as well as haemodynamic parameters: SBP, MAP, DBP, and CO following induction of hypovolaemic shock) and treatment with HG (SpeedM) and CG (QuikClot Combat Gauze). Data are presented as median and interquartile range. For reference range of depicted values, see Table 1. aPTT = activated partial thromboplastin time; CG = Combat Gauze; CO = cardiac output; DBP = diastolic blood pressure; Hb = haemoglobin; Hct = haematocrit; HG = haemostatic gauze; HR = heart rate; MAP = mean arterial pressure; SBP = systolic blood pressure.

In the HG group, Hb (g/dL) levels were T2: 5.4 (5.3, 5.7) and T3: 5.6 (5.3, 5.9), whereas significantly higher values were observed in the CG group (T2: 8.2 [7.4, 9.7] and T3: 7.6 [7.0, 8.2]; .010 < *p* < .020). Hct (%) levels also showed a significant difference (HG group T2: 17 [16, 18] and T3: 17 [16, 18] *vs.* CG group T2: 25 [23, 30] and T3: 23 [21, 25]; T2: .020 < *p* < .050 and T3: .010 < *p* < .020).

No significant differences were found between the groups in terms of platelet count, Quick, and aPTT. In both groups, the animals demonstrated trauma induced coagulopathy.

However, animals in the HG group exhibited significantly lower values for the haemodynamic parameters MAP and DBP (MAP HG group T2: 61 [55, 66] and T3: 60 [52, 64] *vs.* CG group T2: 83 [68, 85] and T3: 77 [73, 80]; *p* < .001 and .005 < *p* < .010; DBP HG group T2: 42 [40, 43] and T3: 39 [33, 47] *vs.* CG group T2: 62 [55, 68] and T3: 64 [56, 67]; .020 < *p* < .050 and .005 < *p* < .010).

SBP showed no significant difference between groups (HG group T2: 94 [81, 96] and T3: 93 [77, 94] *vs.* CG group T2: 94 [85, 106] and T3: 89 [83, 111]; *p* > .20).

### Primary failure and final failure

In the CG group, second application in case of a primary failure was required in two of 11 animals (18%), whereas in the HG group, it was necessary in one of 10 animals (10%). Table 3 presents the corresponding SBP and DBP, as well as the MAP, at the time of failure of the first application (MAP: HG 49 mmHg; CG 37 and 47 mmHg). Following the second application, none of these animals died during the remaining experimental time.

**Table 3:** Blood pressure measurements (systolic blood pressure [SBP], mean arterial pressure [MAP], and diastolic blood pressure [DBP]) at the time of required second application.

Parameter	HG	CG	
SBP – mmHg	89	62	50
MAP – mmHg	49	37	47
DBP – mmHg	34	26	41

Depiction of blood pressure measurements (SBP, MAP, and DBP) at the time of required second application (once in the HG group and twice in the CG group). Failure of the initial application occurred before reaching time point T2 (return to baseline) following shock (T1). None of the animals died during the remaining experiment time (HG SpeedM; CG QuikClot Combat Gauze). CG = Combat Gauze; DBP = diastolic blood pressure; HG = haemostatic gauze; MAP = mean arterial pressure; SBP = systolic blood pressure.

Final failure of the product beyond fifteen minutes after application occurred in the CG group (*n* = 3/11, 27%) at thirty-seven, fifty, and fifty minutes after shock induction (T1). Two of these animals had a MAP ≥60 mmHg (MAP 71 and 78 mmHg), whereas one had a MAP <60 mmHg (MAP 55 mmHg) (Table 4). In the HG group, one animal (1/10, 10%) died fifty minutes after T1, before reaching a MAP ≥60 mmHg (MAP 56 mmHg).

**Table 4:** Time of final failure in the form of uncontrollable bleeding.

Parameter	HG	CG		
*t* – min	57	37	50	50
SBP – mmHg	85	93	84	74
MAP – mmHg	56	78	71	55
DBP – mmHg	40	64	55	39

Time point (min) of final failure in terms of uncontrollable bleeding after shock (T1). Blood pressure values (SBP, MAP, and DBP) at the onset of uncontrollable bleeding (HG SpeedM; CG QuikClot Combat Gauze). CG = Combat Gauze; DBP = diastolic blood pressure; HG = haemostatic gauze; MAP = mean arterial pressure; SBP = systolic blood pressure.

## DISCUSSION

This study compares a halloysite based HG (SpeedM, 7 Å halloysite) with the kaolin based QuikClot CG for the management of arterial haemorrhage in a standardised lethal CFA bleeding model. The findings offer new insights into the applicability and efficacy of halloysite in the emergency treatment of traumatic arterial bleeding, compared with QuikClot CG.

The aforementioned haemorrhage model used to test haemostatic agents is based on the design by Kheirabadi *et al.*^19^^,^^18^ Key factors in this bleeding model include a standardised vascular defect (4.6 mm), a fixed compression pressure of 200 mmHg applied for 5 minutes to the injured site, the use of Doppler ultrasound to monitor post-intervention perfusion, and continuous intravenous heparin administration to ensure comparable anticoagulation status in all animals.

Measurement of post-interventional perfusion serves to exclude thrombotic occlusion following application of the product, an adverse event previously observed with smectite based haemostatic powders such as WoundStat.^20^ In contrast to powdered formulations, the mineral components in both kaolin based CG and halloysite based HG are incorporated into a carrier matrix. Unlike WoundStat, the carrier matrix of both gauzes could be completely removed from the wound post-mortem.

A previous study demonstrating thromboembolic events associated with smectite, showed that kaolin detaches from the carrier matrix but is not considered the direct cause of thrombus formation.^20^ However, Otrocka-Domagała *et al.*^21^ detected kaolin containing thrombotic material in the pulmonary vessels of one animal twenty-four hours after the application of CG.

This study was performed in anticoagulated animals in order to demonstrate the effectiveness of both haemostatic agents in anticoagulated patients as well. In discussions about the efficacy of haemostatic agents, it is often overlooked that anticoagulation in porcine bleeding models is essential owing to the markedly accelerated endogenous coagulation processes in pigs compared with humans. The activity levels of individual factors involved in the intrinsically activated coagulation pathway are significantly higher in pigs than in humans.^22^, ^23^, ^24^ Lechner *et al.*^25^ were able to demonstrate that kaolin based haemostatic agents exhibit measurable haemostatic effectiveness in anticoagulated blood.

The intrinsic coagulation cascade was impaired by the administration of a single dose of 10 000 IU enoxaparin sodium at the beginning of the experiment, followed by a continuous infusion of 500 IU/h throughout the duration of the study. The extrinsic coagulation cascade was affected by trauma induced consumption of coagulation factors as a result of bleeding.

Quick decreased from baseline to the time point of manifest haemorrhagic shock (HG: 97% [86, 111] to 77% [68, 81]; QC: 96% [89, 105] to 89% [82, 99]), whereas aPTT increased after the onset of haemorrhagic shock (HG: 99 seconds [88, 122] to 113 seconds [110, 125]; QC: 101seconds [85, 114] to 127 seconds [109, 151]).

It should be noted that both products were capable of achieving effective haemostasis under coagulopathy. The possible need for a second application of the product following an initial five minute compression phase at 200 mmHg reflects real world conditions in the management of deep and heavily bleeding wounds where soldiers have just two haemostatic dressings in their individual medical equipment.

Such a second application was necessary in the HG group once (*n* = 1; 10%; MAP 49 mmHg) and in the CG group twice (*n* = 2; 20%; MAP 37 and 47 mmHg, respectively).

Following a second application, no additional product was applied in the event of recurrent bleeding during the subsequent course of the study. This was defined as final failure, representing a scenario analogous to the pre-hospital transportation phase, in which no further interventions are usually possible.

In the HG group, one animal (*n* = 1; 10%) experienced final failure fifty minutes after shock induction (MAP = 56 mmHg). In contrast, in the CG group, three animals (*n* = 3; 33%) died, one at thirty-seven minutes and two at fifty minutes post-shock, with MAPs of 78, 71, and 55 mmHg, respectively.

It should be considered that under permissive hypotensive conditions (target MAP ≈50–60 mmHg), as recommended by the European guideline on the management of major bleeding and coagulopathy following trauma, only one animal per group would have died (HG 56 mmHg; CG 55 mmHg).^26^

In a retrospective analysis of registry data from the German Society for Trauma Surgery (TraumaRegister), Imach *et al.*^27^ demonstrated that over 60% of trauma patients, who should be managed with permissive hypotension according to the aforementioned European guideline, presented with a SBP >90 mmHg on hospital admission.

Consequently, an emergency haemostatic agent should offer a therapeutic safety margin that tolerates transient hypertensive episodes, with temporary SBP peaks >80 mmHg during transport or in cases of inadequate sedation.

In the CG group, two animals died above the blood pressure cutoff for permissive hypotension (MAP ≈65 mmHg): one at MAP = 71 mmHg and another at MAP = 78 mmHg.

It would be advisable to expand the criteria for an ideal haemostatic agent as defined by Pusateri *et al.*,^28^ not only requiring that arterial and venous bleeding be controlled within two minutes but also providing therapeutic safety at blood pressure levels exceeding those of permissive hypotension.

### Conclusion

This study demonstrates halloysite based gauze as an alternative to kaolin based gauze. The distinctive physicochemical properties of halloysite, such as its nanotubular morphology characterised by a hollow lumen and oppositely charged inner and outer surfaces, a higher effective surface area, enhanced adsorption capacity, and the ability to encapsulate and controllably release active agents, may offer new opportunities in the development of advanced haemostatic materials.

The relatively small number of test animals (*n* = 21) may limit the statistical power of this study. To confirm and expand upon these findings, future investigations with larger sample sizes and additional traumatic bleeding models are warranted.

## ETHICAL STATEMENT

The study was approved by the Authority of Public Health and Consumer Protection of the city of Hamburg, Germany, with the approval number 112/14.

## FUNDING

None.

## CONFLICTS OF INTEREST

Daniel Hinck and Sebastian Debus have received consultant honoraria from SpeedMineral, Neubrandenburg.
